# Psychological Impact of Alopecia Areata

**DOI:** 10.1155/2020/8879343

**Published:** 2020-12-24

**Authors:** Suchana Marahatta, Sudha Agrawal, Baikuntha Raj Adhikari

**Affiliations:** Department of Dermatology and Venereology, Department of Psychiatry, B. P. Koirala Institute of Health Sciences, Dharan, Nepal

## Abstract

**Introduction:**

Alopecia areata is one of the commonest causes of nonscarring alopecia. Since hair is a vital part with very high cosmetic concern, hair loss might have a significant negative impact on patient's life. Hence, we aimed this to investigate depression and anxiety in patients with alopecia areata.

**Methods:**

In this cross-sectional study, a total of 75 consecutive eligible patients of alopecia areata were interviewed over one-year period in the dermatology outpatient department. We recorded the relevant history and examination details in the present proforma. Nepali versions of Beck Depression Inventory and Beck Anxiety Inventory were used for the assessment of depression and anxiety, respectively. Data analysis was done with Statistical Package for the Social Sciences version 11.5.

**Results:**

Among 75 patients, the prevalence of depression and anxiety were 66.7% and 73.3%, respectively, with median depression score = 5 (IQR = 0.0–10.0) and median anxiety score = 5 (IQR = 0.0–11.0). Out of all depressed patients, 82.0% had minimal and 18.0% had moderate depression. However, none of them had severe depression. Likewise, out of all patients with anxiety, 89.0% had mild and 11.0% had moderate anxiety, but none of them had severe anxiety.

**Conclusion:**

Anxiety and depression are common psychological problems in patients with alopecia areata. Because of their direct impact on treatment outcome, we, treating dermatologist, must be aware of them, and we should counsel our patients for consultation with the psychiatrist on time for their maximum benefit.

## 1. Introduction

Alopecia areata (AA) is a nonscarring hair disorder with a frequency ranging from 0.7% to 3.8% of patients attending dermatology clinics with a lifetime risk of 1.7% [1, 2]. Because of the important cosmetic and communicational role of human hair, one can expect significant psychological distress in person with partial or complete hair loss [3]. Though it is a benign condition, it can cause detrimental psychological impacts on individual's life. A few past studies have reported that AA really affects the quality of life of the affected individual [4, 5]. In one of the studies, as many as 61% of the AA participants had depression [6]. Moreover, if the negative psychological impacts are not addressed on time, it may further worsen the disease condition [7], and hence there can be a vicious cycle as well. A recent study had also proved a bidirectional association between AA and depression [8]. However, some evidence has not supported this relation [9]. So, we still need more studies on this matter.

Though not life-threatening, this visible disorder can directly affect the way how people think about themselves and others. Despite significant psychological impact on mental well-being, AA still remains under-researched. Though there are few studies on this topic around the globe, we could not find any published data from Nepal. As there could be a bidirectional association between AA and psychological comorbidities, if it is not addressed on time, we may not get optimum treatment response. Knowing the associate psychological comorbidities, we can plan for timely intervention to minimize them and to maximize the treatment outcome. Hence, we conducted this study to investigate depression and anxiety in patients with AA, in a tertiary referral hospital of eastern Nepal.

## 2. Materials and Methods

We conducted this cross-sectional study in the outpatient dermatology clinic of B. P. Koirala Institute of Health Sciences, Dharan, Nepal, over one-year period (from August 2015-July 2016). This is a part of a study entitled “Alopecia areata and autoimmunity: considering its association with thyroid disorder and its psychological impacts.” Ethical clearance was obtained from the Institutional Review Committee of BPKIHS (code no: IRC/584/015). All subjects with new onset of AA but without other dermatological problems who attended the dermatology outpatient department of B. P. Koirala Institute of Health Sciences were included in the study. However, those not willing to participate in the study, age <14 years, known cases of underlying psychiatric illness, pregnant women, known case of thyroid disorders, and subjects on treatment for AA were excluded from the study. Its further detail was mentioned in the previous study [10].

After written consent, a detailed history was taken, and examination was done. The relevant history and examination findings were noted in the pre-set proforma. Diagnosis of AA was made clinically by the consultant with more than twenty years of experience in dermatology. In case of diagnostic dilemma, biopsy was performed for confirmation. AA severity was graded according to the following: (i) AA severity grading system proposed by Kavak et al. into mild, moderate, severe, and snake-like pattern (ophiasis) and [11] (ii) Severity of Alopecia Tool (SALT) score was calculated as proposed by Olsen et al. [12]. For the assessment of depression and anxiety, Nepali version of Beck Depression Inventory and Beck Anxiety Inventory were used, respectively [13, 14].

### 2.1. Assessment of Depression

Nepali version of Beck Depression Inventory (BDI) comprises 21 questions. Rating of BDI was done as follows: no depression, score 0–9; mild-moderate depression, score 10–18; moderate-severe depression, score 19–29; and severe depression, score 30–63 [13].

### 2.2. Assessment of Anxiety

Nepali version of Beck Anxiety Inventory (BAI) also comprises 21 questions. The rating of BAI was done as follows: very low anxiety, score 0–21; moderate anxiety, score 22–35; and severe anxiety, score >36 [14].

## 3. Statistical Analysis

Data were entered into MS Excel 2010. After cleaning the data, they were exported to licensed SPSS version 11.5. For the descriptive statistics, % proportion, mean, median, standard deviation, and interquartile range (IQR) were calculated, and for inferential statistics, Chi-square test and Kruskal–Wallis tests were applied at 95% CI and *P* = 0.05.

### 3.1. Results

A total of 75 consecutive patients of AA were interviewed over one-year period. The mean age of the patients was 29.40 ± 9.90 years, with male (53.3%) and female (46.7%) ratio being 1.14 : 1. The median disease duration was 2 months (IQR = 1–5). Median number of lesions was 2 (IQR = 1–4). Median Severity of Alopecia Tool (SALT) score was 24.7 (IQR = 9.6–57.9). Its detail has been published in the previous study [10].

The prevalence of depression among AA patients was 66.7% with median depression score = 5 (IQR = 0.0–10.0). Mild to moderate depression was present in 41 (82%) AA patients, and moderate to severe depression was present in 9 (18.0%) AA patients. However, severe depression was not found in any of the patients.

Prevalence of anxiety was 73.3% with median anxiety score = 5 (IQR = 0.0–11.0). Maximum of them 49 (89.0%) had very low anxiety, and moderate anxiety was present in only 6 (8.0%) AA patients. However, severe anxiety was not present in any of the patients.

### 3.2. Relation between Psychological Impact and Severity of Alopecia Areata

1. Relation of depression with severity of AA: though the severity of depression got increased along with increased SALT score, there was no statistical significance (*P*=0.49) (Table 1). Similarly, though the severity of depression was more in individuals with higher severity grade of AA (*P* < 0.05), its clinical significance is not relevant (Table 2).
2. Relation of anxiety with severity of AA: there was no relation between anxiety and SALT score (*P*=0.12) (Table 3). Though moderate anxiety was more in individuals with AA severity grade 2, as compared to grade 1, we could not find any relation between AA severity grading and anxiety severity (*P*=0.14) (Table 4).

## 4. Discussion

In our study, among 75 AA patients, the prevalence of depression was 66.7% with median depression score = 5 (IQR = 0.0–10.0). Similarly, the prevalence of anxiety was 73.3% with and median anxiety score = 5 (IQR = 0.0–11.0). Among depressed ones, 82.0% had minimal and 18.0% had moderate depression, while none of them had severe depression. Similarly, 89.0% of AA patients had mild and 11.0% had moderate anxiety, while none of them had severe anxiety.

Hair loss, though mostly a benign condition, can cause severe adverse psychological impact on the individual's life because of its cosmetic importance. Williamson et al. reported that the mean Dermatological Life Quality Index (DLQI) score of the participants with alopecia was comparable to that of severe psoriasis and atopic dermatitis. Clinical depression and loss of self-confidence were present in 74% and 30% of the participants, respectively. A thorough analysis revealed that lowered self-esteem was reported in approximately 23% of patients [15]. Likewise, according to an Iranian study, 58% of AA patients believed that the disease has a major effect on their lives and self-concept was affected in 53%. Likewise, 40% of the participants thought that their illness would be permanent [16]. Similarly, in another study, 61% of the participants were depressed. More than half of the depressed patients had moderate to severe depression. Therefore, they recommended that Quality of Life (QoL) impairment and mood disorders should be considered and managed on time in all individuals with AA [6]. Like these studies, we also found higher prevalence of depression (66.7%) and anxiety (73.3%) in our patients. Comparable were the findings in another study where either depression or anxiety was present in 65.9% of adults with AA [17]. However, unlike our findings, Karia et al. had reported very low anxiety (4%) and depression (18%) in AA patients [18]. Similarly, another study done by using the similar assessment tools like ours (i.e., BDI and BAI) has also reported that there were no differences in depression and anxiety levels between AA participants and controls [9]. Hence, the topic is still debatable with inconsistent results.

A recent systematic review had also reported a significant psychological impact by AA. The authors could find a total of 28 articles focused on this issue. Out of them, 12 had assessed the relation between psychiatric problems and AA. Nine of them revealed higher existence of anxiety, depression, and other psychiatric morbidities in AA patients compared to the controls. Thus, the authors have recommended that psychological impacts and comorbidities must be addressed while treating AA patients [19]. Williamson et al. reported slightly higher prevalence of depression (74%) as compared to our study. However, this could be because the patients in this study were enrolled through a patient support group and they had very long median duration (138 months) of AA as compared to ours. Moreover, the tool they had used to detect depression was just a screening questionnaire. Hence, there might be some false-positive cases as well [15]. The association between AA and depression has further been highlighted by the bidirectional study. In this study, authors have demonstrated AA increases the chance of major depressive disorder by 34% (hazard ratio, 1.34; 95% CI, 1.23–1.46; *P* < 0.001). Likewise, depressive disorders can also increase the risk of AA by 90% (hazard ratio, 1.90; 95% CI, 1.67–2.15; *P* < 0.001) [8]. Hence, we must be vigilant for the psychological comorbidities in AA individuals to minimize a vicious cycle between them.

Tan and colleagues found that 82% of respondents with extensive AA exhibited significantly higher psychological distress throughout their lives than those with limited AA. The findings suggested that elevated depression, anxiety, and stress levels are more prone in individuals with severe types of AA, compared to the milder ones. Therefore, the authors concluded that, as the severity of AA increases, so does the likelihood of individuals developing psychological comorbidities [1]. However, in our study, we could not establish any relation between the severity of AA and psychological status (depression and anxiety) of AA patients. This could be because of lesser sample size, shorter duration of disease, and lesser number of patients with severe AA in our study. Likewise, another important reason could be because of direct and easy access to dermatologist in Nepal. Hence, patient presented to us immediately with less severe disease and less psychological impacts. Similarly, the questionnaire used by Tan et al. was more specific to hair loss which might be more relevant and specific than the tools we had used [1]. Similar to our study, Matzer et al. [7] and Cartwright et al. [20] revealed that AA severity did not influence the development of depression and anxiety. It could be because of the difference in coping capacity of the individuals with stressful life events. Likewise, AA affecting more noticeable or visible parts such as frontal scalp, and facial hair may cause more depression and anxiety even if hair loss is less severe.

There is evidence that, besides the medical therapies, hypnotherapy is also effective in the treatment of the AA [21, 22]. However, another recent study could not establish the role of hypnotherapy for the clinical outcome in AA [23]. But these studies have concluded that hypnotherapy has significant positive impacts on psychological well-being of AA patients. These further strengthen and highlight the importance of psychological impact on AA. Also, in our study, a significant percentage of AA patients had depression (66.7%) and anxiety (73.3%). All dermatologists may not be paying enough attention on the psychological and emotional issues associated with AA. With less attention, we might be overlooking the patient's real overall health status. Therefore, effective treatment of the AA patients should also include the assessment and necessary management of psychological comorbidities for optimal outcome and holistic care of the patient. However, we have some limitations in our study. We had small sample size. A case-control or cohort study design with longer follow-up of the patients would have given better idea about depression and anxiety among AA patients.

## 5. Conclusion

In Nepal, anxiety and depression are common psychological problems in AA patients. Hence, while dealing with AA, we, treating dermatologists, must pay attention to these aspects. However, in this study, we could not establish a relation between AA severity and psychological impacts (anxiety and depression). Hence, cohort studies with larger sample size might give a clear picture on this matter.

## Figures and Tables

**Table 1:** Relation between depression and SALT score.

Depression severity	Median SALT score	*P* value
No depression (*n* = 25)	21.6	0.49 (Kruskal–Wallis test)
Mild-moderate depression (*n* = 38)	23.4	
Moderate-severe depression (*n* = 9)	35.0	

**Table 2:** Relation between depression and alopecia areata severity grading.

Depression severity	Alopecia areata severity grading			*P* value
	Grade 1	Grade 2	Ophiasis	
No depression (*n* = 25)	7 (28%)	15 (60%)	3 (12%)	<0.05 (Chi-square test)
Mild-moderate depression (*n* = 41)	16 (39%)	11 (27%)	14 (34%)	
Moderate-severe depression (*n* = 9)	1 (11%)	5 (56%)	3 (33%)	

**Table 3:** Relation between anxiety and SALT score.

Anxiety severity	Median SALT score	*P* value
No anxiety (*n* = 20)	19.9	0.12 (Kruskal–Wallis test)
Very low anxiety (*n* = 48)	33.4	
Moderate anxiety (*n* = 2)	18.0	

**Table 4:** Relation between anxiety and alopecia areata severity grading.

Anxiety severity	Alopecia areata severity grading			*P* value
	Grade 1	Grade 2	Ophiasis	
No anxiety (*n* = 20)	6 (30%)	12 (60%)	2 (10%)	0.14 (Chi-square test)
Very low anxiety (*n* = 49)	16 (33%)	15 (30%)	18 (37%)	
Moderate anxiety (*n* = 6)	2 (33%)	4 (67%)	0 (0)	

## Data Availability

Data were deposited in a repository and can be provided by the corresponding author upon request.
